# The psychiatric effects of varenicline on patients with depression

**DOI:** 10.1186/1753-6561-9-S1-A31

**Published:** 2015-01-14

**Authors:** EYH Yeung, S Long, BL Bachi, J Lee, Y Chao

**Affiliations:** 1 College of Medical and Dental Sciences, The University of Birmingham, Birmingham, United Kingdom

## Background

Varenicline is currently the most effective smoking cessation medication. Pre-marketing clinical trials excluded participants with psychiatric disorders, such as major depressive disorders. This study investigated the psychiatric effects of varenicline among patients with depression.

## Methods

On 18 December 2012, a systematic search was performed using Medline with the following search terms: 1) varenicline and 2) depression. The search was limited to English articles, case reports, and original clinical studies. From the 58 retrieved documents, 15 articles were used in this review.

## Results

The first case report on the effects of varenicline on patients with depression was published in June 2008. A man experienced an acute exacerbation of depressive symptoms, which resolved after he stopped his varenicline treatment. [1] Since then, there were 8 other case reports that described exacerbation of psychiatric symptoms in patients with depression taking varenicline [2-9]. Two of those case studies suggested the use of sertraline [7] and bupropion [8] to treat exacerbation of depressive symptoms associated with varenicline. In contrast, varenicline was shown to improve the affective symptoms of a smoker who developed depression and suicidal tendencies during previous cessation attempts [10]. There were 3 observational studies on patients with depression taking varenicline: 1) a one-year follow-up study on 112 smokers showed an association between increased Beck Depression Inventory score and continued smoking after 12 weeks of varenicline [11]; 2) an open-labelled study showed significant improvement in mood in 110 outpatient smokers with persistent depressive symptoms [12]; and 3) A smoking cessation trial on 217 varenicline users showed that depressive symptoms at the time of varenicline initiation (measured by Patient Health Questionnaire-2) were associated with suicidal ideation. [13] There were 2 clinical trials on patients with depression taking varenicline, with both of them showing worsening of psychiatric symptoms. [14,15] Neither of the trials were placebo-controlled.

## Conclusions

Despite some inconsistencies, the findings suggested that varenicline could worsen psychiatric symptoms in patients with depression. Clinicians should be advised to closely monitor patients with a history of depression on varenicline, although there were no studies on how to treat those patients. Bias and uncontrolled confounders potentially affected previous studies, and thus, a double-blinded placebo-controlled trial is needed to demonstrate the efficacy and side effects of varenicline on patients with depression.
